# Bilateral acute macular neuroretinopathy following COVID‐19 infection

**DOI:** 10.1111/aos.14913

**Published:** 2021-05-26

**Authors:** Clarice Giacuzzo, Chiara M. Eandi, Aki Kawasaki

**Affiliations:** ^1^ Hôpital Ophtalmique Jules Gonin Fondation Asile des Aveugles Lausanne Switzerland; ^2^ Department of Surgical Science University of Torino Torino Italy; ^3^ Department of Biology and Medicine University of Lausanne Lausanne Switzerland

Editor,

A 23‐year‐old woman had fatigue, nasal congestion, headache, vertigo and sweating for two weeks without fever. The next day, she noticed photopsias which evolved to several paracentral scotomas in both eyes. Ophthalmologic examination in the emergency room revealed best‐corrected visual acuity of 20/20 in both eyes. Ishihara colour vision testing revealed dyschromatopsia only in the left eye. Pupillary reaction, ocular motility, biomicroscopy and funduscopy were within normal limits. Spectral‐domain optical coherence tomography (SD‐OCT, Heidelberg, Germany) of the macula and the optic nerve of both eyes were normal. Because of her preceding systemic symptoms, PCR testing for SARS‐CoV‐2 using nasopharyngeal swab was performed, and she tested positive. MR imaging of the brain was normal, and serologic testing of blood and cerebrospinal fluid was done for various infectious and inflammatory disorders, and the result was negative. No specific diagnosis or treatment was proposed. Ten days after presentation in the emergency room, the patient had a painful vesicular eruption on the upper lip. This was diagnosed as herpes labialis (herpes simplex‐1, HSV‐1) and treated with topical antiviral cream with a positive outcome in 6 days.

**Fig. 1:**
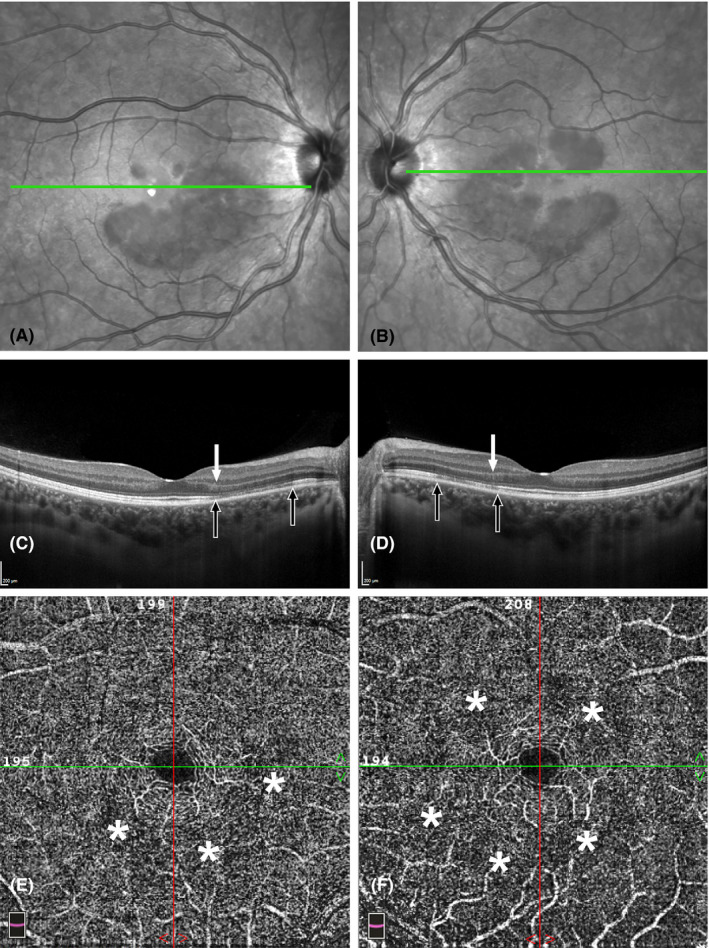
(A,B) Near‐infrared imaging of right eye (A) and left eye (B) shows multiple hyporeflective lesions in the paracentral macula in both eyes. The small lesions have a petaloid shape, but the majority of lesions are large and confluent, almost forming a ring around the fovea. (C, D) Spectral‐domain optical coherence tomography of right eye (C) and left eye (D) at the level of the green line in Figure A, B, shows interruption of the ellipsoid zone and the interdigitation zone (black arrows). There are also hyperreflective changes (white arrow) within the outer nuclear layers. (E, F) Optical coherence tomography angiography of the right eye (E) and left eye (F) shows multiple areas of decreased vascular flow signal (asterisks) at the level of the deep capillary plexus corresponding to the lesions visible in the near‐infrared reflectance imaging.

After three weeks, all symptoms spontaneously disappeared except the visual disturbance for which she was then referred for neuro‐ophthalmologic consultation. The paracentral scotomas had become semi‐transparent but were otherwise unchanged. The dyschromatopsia of the left eye was cured; funduscopy was still normal. Visual field testing with Amsler grid and Octopus 900 perimetry, 30‐degree field analysis (Haag Streit, Bern, Switzerland) showed several paracentral scotomas within the central fifteen degrees of both eyes. Spectral‐domain optical coherence tomography (SD‐OCT) now revealed disruption of the ellipsoid and interdigitation zones with hyperreflectivity within the outer nuclear layers in the paracentral macula. Near‐infrared reflectance (NIR) imaging showed large, bilateral confluent hyporeflective lesions and smaller petaloid‐shaped lesions around the fovea, corresponding to the region of OCT abnormalities. (Fig. 1A–D). Fluorescein angiography and indocyanine green angiography were normal in both eyes. Optical coherence tomography angiography (OCT‐A, Angiovue RTx 100, Optovue, Inc, Fremont, CA, USA), however, showed decreased vascular flow signal at the level of the deep capillary plexus (DCP) in the region of the OCT and NIR abnormalities (Fig. 1E, F). A final diagnosis of bilateral acute macular neuroretinopathy (AMN) was made. Given her excellent visual acuity, no specific treatment was recommended. One month later, the examination and ancillary testing did not show any remarkable changes.

In a previous review of 101 cases of AMN (Bhavsar et al. 2016), almost half were associated with a preceding respiratory or influenza‐like illness. Our patient had preceding flu‐like symptoms from COVID‐19, and shortly after noticing the visual disturbance, she had a herpes labialis eruption. We believe this was either concomitant infection with SARS‐CoV‐2 or reactivation by SARS‐CoV‐2. The published literature does not implicate HSV‐1 virus with AMN.

The pathophysiology of AMN is a non‐inflammatory vaso‐occlusive disorder of retinal capillaries. Optical coherence tomography (OCT) and OCTA changes related to capillary vasculopathy have been reported in the DCP and/or choriocapillaris (Casalino et al. 2019), (Fawzi et al. 2012). The typical fundus abnormality of AMN is one or more wedge‐shaped, well‐delineated lesions pointing to the fovea (Bhavsar et al. 2016). AMN following COVID‐19 infection is rare. Two previous published cases (Gascon et al. 2020; Virgo & Mohamed 2020) reported small, focal petaloid lesions in one affected eye. However, our patient demonstrated unusually large, confluent lesions in both eyes, which suggested a large area of retinal pathology. We wonder whether endotheliopathy due to direct SARS‐CoV‐2 infection predisposes to greater retinal ischaemia and larger lesions of AMN (Iba et al. 2020). Systematic ophthalmologic examination of patients with coronavirus disease may clarify the prevalence and clinical profile of AMN associated with COVID‐19.Open Access Funding provided by Universite de Lausanne.
